# Construction of a Nomogram predictive model for post-discharge psychosomatic review of psychiatric liaison consultation patients based on medical record data

**DOI:** 10.3389/fpsyt.2023.1171741

**Published:** 2023-07-12

**Authors:** Liu Yanwen, Li Mei, Zhang Wenwen, Jing Huihui, Lu Hongbin, Wang Ying, Liu Ning, Han Le, Han Xueyang, Zou Xue

**Affiliations:** ^1^Department of Adolescent Mental Health, Mental Hospital, Xi’an International Medical Center, Hospital of Northwest University, Xi’an, China; ^2^Department of Psychosomatic Medicine, Mental Hospital, Xi’an Physical Education University, Xi’an, China; ^3^Department of Psychosomatic Medicine, Mental Hospital, Xi’an International Medical Center, Hospital of Northwest University, Xi’an, China

**Keywords:** consultation-liaison psychiatry, Nomogram predictive model, mental health, psychiatric disorders, follow-up

## Abstract

Epidemiological studies have shown that almost all physical illnesses coexist with psychiatric disorders or psychological problems, and the severity of mental illness is positively correlated with the duration and severity of physical illness. Liaison consultations are valuable in identifying and treating psychiatric disorders, but the rate of psychiatric follow-up after consultation is low in outpatients. This study aimed to investigate the factors influencing post-discharge psychosomatic follow-up visits in patients undergoing psychiatric liaison consultation in general hospitals and construct a Nomogram prediction model for patients’ post-discharge psychosomatic follow-up visits. Medical record data of inpatients who received psychiatric liaison consultations at Xi’an International Medical Center Hospital in China from September 2019 to September 2020 were analyzed. Lasso regression and multivariate logistic regression analyses were conducted to screen independent influences on the occurrence of post-discharge psychosomatic follow-ups in patients undergoing psychiatric liaison consultations. Risk prediction column line graphs were constructed using R software, and the models were evaluated. Of the 494 inpatients who received psychiatric liaison consultations, 115 patients (23.279%) (mean age = 54.8 years) went for post-discharge psychosomatic follow-up, while 379 patients (mean age = 59.3 years) had no record of psychosomatic follow-up. Furthermore, occupation, interval.time, diagnosis, out.antipsychotics, and recommendations.followup were independent factors influencing post-discharge psychosomatic follow-up. The model accurately predicted post-discharge psychosomatic follow-up behavior of inpatients who received psychiatric liaison consultations. Lastly, the clinical decision curve analysis showed that the model had good validity for clinical application. Patients who received a psychiatric liaison consultation with a ≤ 10-day interval between admission to the hospital and application for consultation, were discharged with prescribed medication, and had a clear written medical order for a follow-up consultation had an increased probability of psychosomatic follow-up after discharge.

## 1. Introduction

Consultation-liaison psychiatry (CLP) is an important development in the area of mental health. It is a well-established treatment model in several developed countries (1), whereas related research lags in China (2). There is a significant gap between CLP services in China and abroad, which is reflected in the inadequacy of service models as well as the irregular consultation and referral rate. This gap can be attributed to the relatively short history of CLP development, insufficient resources for psychiatric/psychological treatment, and sociocultural influences in China. The psychiatric liaison consultations in general hospitals in China primarily involve a non-psychiatrist submitting a request for consultation to a psychiatrist, who then conducts consultation with the patient. However, this form of CLP cannot meet the patients’ demand for psychiatric/psychological services. In practice, in Chinese hospitals, the psychiatric follow-up rate of consultation patients after consultation is much lower than that of outpatients. There are few quantitative studies on CLP in China, and the reported psychiatric consultation rates (1.0 to 2.3%) are much lower than those reported abroad (2.6 to 3.3%) (3). The development of psychiatric symptoms in some patients is associated with multiple factors and requires multiple consultation sessions and psycho-behavioral treatment. Although the psychiatric problems of patients are identified in the consultation, effective patient follow-up and follow-up treatment are not achieved, which is not only detrimental to patients but is also a waste of consultation resources; therefore, it is necessary to improve the follow-up rate of consultation patients to increase the value of consultation work. The Nomogram prediction model is an intuitive and convenient tool that integrates and assigns weights to influencing factors to estimate an outcome (4). This study aimed to investigate the factors influencing post-discharge follow-up visits in patients who received psychiatric liaison consultations in general hospitals in China and develop a Nomogram prediction model for patients’ post-discharge follow-up, providing a reference for the clinical assessment of patients’ need for follow-up and how to promote proactive follow-up in patients.

## 2. Subjects and methods

### 2.1. Design

This is a retrospective case study aimed at constructing a Nomogram prediction model for factors influencing patients’ post-discharge psychosomatic follow-up visits.

### 2.2. Time and place

Medical record data of inpatients who received psychiatric liaison consultations at Xi’an International Medical Center Hospital from September 2019 to September 2020 were collected.

### 2.3. Subjects and selection criteria

#### 2.3.1. Inclusion criteria

Inpatients at Xi’an International Medical Center Hospital who received psychiatric liaison consultations between 1 September 2019 and 1 September 2020, including inpatients in emergency holding rooms.

#### 2.3.2. Exclusion criteria

Inpatients whose cases did not require follow-up consultations.

Finally, a total of 494 medical records of patients who received psychiatric liaison consultations at Xi’an International Medical Center Hospital from September 2019 to September 2020 were retrospectively collected.

### 2.4. Methods

#### 2.4.1. Data collection

(1) Basic patient information: age, interval.time (interval between admission to hospital and application for consultation), department (hospital department), t.time (hospitalization interval), residence (Xi’an, non-Xi’an), occupation (farmer, technical staff, retiree, student, other); (2) consultation records: reason (reason for consultation: physical symptoms that are difficult to explain or poorly treated, with or without anxiety or depression, excluding psychiatric disorders; patients with anxiety or depressive manifestations, not excluding psychiatric disorders; sleep disorders; previous history of anxiety and depression; hallucinations, delusions, behavioral arousal disorders; previous history of other psychiatric disorders); diagnosis (anxious depressive state; sleep disorders; organic mental disorders; stress-related disorders; schizophrenia or paranoid psychosis); treatment (drugs, combination therapy); in.antipsychotics (whether psychotropic medication was used during hospitalization); out.antipsychotics (whether medication was taken after discharge); recommendations.followup (whether medical advice recommended a follow-up visit to the psychosomatic department); (3) follow-ups (whether patients made the follow-up visit).

#### 2.4.2. Follow-up records

**FIGURE 1:**
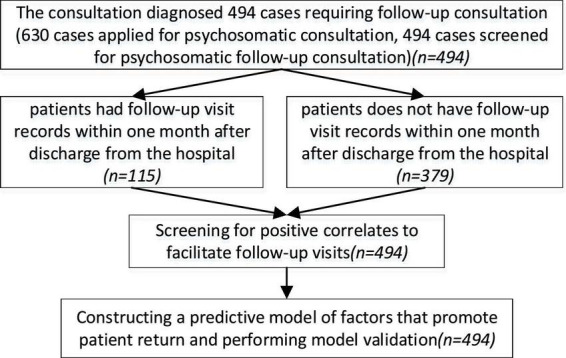
Experimental flow chart.

Obtained the patients’ visit records for 1 month after discharge from the hospital, as well as their medical history records and consultation records. The research process is shown in Figure 1.

### 2.5. Main observation indicators

The Lasso (least absolute shrinkage and selection operator) regression and multivariate logistic regression analyses were conducted to screen for factors influencing patients’ post-discharge psychosomatic follow-up visits. Risk prediction column line graphs were constructed using R software, and the models were evaluated.

### 2.6. Statistical analysis

Measurement data were described as $\bar{x}\pm s$, and count data were represented as percentages and composition ratios, which were compared using analysis of variance (ANOVA) and chi-square test, with *p* < 0.05 being considered statistically significant. SPSS 22.0 software was used to process the data.

The Lasso regression was performed to screen the factors influencing patients’ post-discharge psychosomatic follow-up, and binary logistic regression was used to construct a predictive model for patients’ post-discharge psychosomatic follow-up. The model was validated internally and externally by plotting the receiver operating characteristic (ROC) curve and calculating the area under the curve. The prediction model was evaluated through calibration curve analysis and clinical decision curve analysis.

## 3. Results

### 3.1. Comparison of the general conditions of patients

Of the 494 patients who received psychiatric liaison consultations at Xi’an International Medical Center Hospital from September 2019 to September 2020, 115 patients went for follow-ups in the psychosomatic department, and 239 patients did not go for a follow-up visit. As shown in Table 1, there were significant differences between the two groups of patients in terms of occupation, interval.time, diagnosis, out.antipsychotics (*p* < 0.05), and recommendations.followup (*p* < 0.001).

**TABLE 1:** Comparison of the general conditions of patients in the two groups.

Variables	Total (*N* = 494)	Follow-up visits (*n* = 115)	No repeat visits (*n* = 379)	*p*
Age, mean ± SD	58.3 ± 15.5	54.8 ± 16.1	59.3 ± 15.2	0.006
**Residence, *n* (%)**				0.769
Xi’an	386 (78.1)	91 (79.1)	295 (77.8)	
non-Xi’an	108 (21.9)	24 (20.9)	84 (22.2)	
**Occupation, *n* (%)**				**0.003**
Farmers	127 (25.7)	36 (31.3)	91 (24)	
Technical staff	57 (11.5)	13 (11.3)	44 (11.6)	
Retirees	86 (17.4)	12 (10.4)	74 (19.5)	
Students	6 (1.2)	5 (4.3)	1 (0.3)	
Other	218 (44.1)	49 (42.6)	169 (44.6)	
T.time, mean ± SD	13.5 ± 14.5	10.6 ± 10.8	14.4 ± 15.4	0.014
**Reason, *n* (%)**				0.133
Physical symptoms that are difficult to explain or poorly treated, with or without anxiety or depression, excluding psychiatric disorders	103 (20.9)	15 (13)	88 (23.2)	
Patients with anxiety or depressive manifestations, not excluding psychiatric disorders	179 (36.2)	47 (40.9)	132 (34.8)	
Sleep disorders	106 (21.5)	27 (23.5)	79 (20.8)	
Previous history of anxiety and depression	51 (10.3)	16 (13.9)	35 (9.2)	
Hallucinations, delusions, behavioral arousal disorders	37 (7.5)	7 (6.1)	30 (7.9)	
Prior history of other psychiatric disorders (schizophrenia, bipolar disorder)	18 (3.6)	3 (2.6)	15 (4)	
**Interval.time, *n* (%)**				**0.003**
≤ 5 days	332 (67.2)	87 (75.7)	245 (64.6)	
6 to 10 days	93 (18.8)	24 (20.9)	69 (18.2)	
11 to 15 days	31 (6.3)	3 (2.6)	28 (7.4)	
> 15 days	38 (7.7)	1 (0.9)	37 (9.8)	
**Diagnosis, *n* (%)**				**0.018**
Anxious depressive state	347 (70.2)	91 (79.1)	256 (67.5)	
Sleep disorders	50 (10.1)	11 (9.6)	39 (10.3)	
Organic mental disorders	61 (12.3)	6 (5.2)	55 (14.5)	
Stress-related disorders	21 (4.3)	2 (1.7)	19 (5)	
Schizophrenia or paranoid psychosis	15 (3.0)	5 (4.3)	10 (2.6)	
**Treatment, *n* (%)**				0.088
Drugs	337 (68.2)	71 (61.7)	266 (70.2)	
Combination therapy	157 (31.8)	44 (38.3)	113 (29.8)	
**In.antipsychotics, *n* (%)**				0.101
Yes	404 (81.8)	100 (87)	304 (80.2)	
No	90 (18.2)	15 (13)	75 (19.8)	
**Out.antipsychotics, *n* (%)**				**0.002**
Yes	217 (43.9)	65 (56.5)	152 (40.1)	
No	277 (56.1)	50 (43.5)	227 (59.9)	
**Recommendations.followup, *n* (%)**				**< 0.001**
Yes	127 (25.7)	106 (92.2)	21 (5.5)	
No	367 (74.3)	9 (7.8)	358 (94.5)	
**Department, *n* (%)**				0.051
Neurology	68 (13.8)	15 (13)	53 (14)	
Nephrology	10 (2.0)	2 (1.7)	8 (2.1)	
Endocrinology	5 (1.0)	2 (1.7)	3 (0.8)	
Rehabilitation	35 (7.1)	7 (6.1)	28 (7.4)	
Rheumatology and immunology	7 (1.4)	4 (3.5)	3 (0.8)	
Digestive surgery	13 (2.6)	1 (0.9)	12 (3.2)	
Geriatrics	14 (2.8)	4 (3.5)	10 (2.6)	
Ear, nose, and throat	5 (1.0)	1 (0.9)	4 (1.1)	
ICU	8 (1.6)	3 (2.6)	5 (1.3)	
Urology	1 (0.2)	0 (0)	1 (0.3)	
Emergency ward	9 (1.8)	2 (1.7)	7 (1.8)	
Cardiology	86 (17.4)	29 (25.2)	57 (15)	
Hematology	3 (0.6)	0 (0)	3 (0.8)	
Thoracic surgery	4 (0.8)	0 (0)	4 (1.1)	
Nail and breast surgery	6 (1.2)	4 (3.5)	2 (0.5)	
Gastroenterology	71 (14.4)	16 (13.9)	55 (14.5)	
Respiratory medicine	51 (10.3)	10 (8.7)	41 (10.8)	
Oncology	25 (5.1)	2 (1.7)	23 (6.1)	
Gynecology	2 (0.4)	1 (0.9)	1 (0.3)	
Orthopedics	30 (6.1)	6 (5.2)	24 (6.3)	
Neurosurgery	27 (5.5)	2 (1.7)	25 (6.6)	
Cardiac surgery	14 (2.8)	4 (3.5)	10 (2.6)	

The bold values in the first column are the category names after classifying the patient’s relevant information data. The specific meaning is explained in section 2.4.1.

### 3.2. Factors influencing patients’ post-discharge follow-up

**FIGURE 2:**
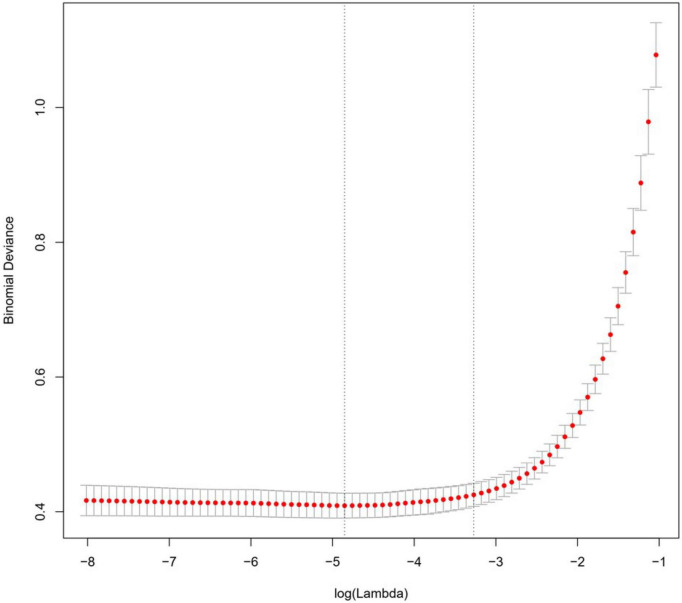
Lasso regression analysis of screening variables: lambda optimal value.

**FIGURE 3:**
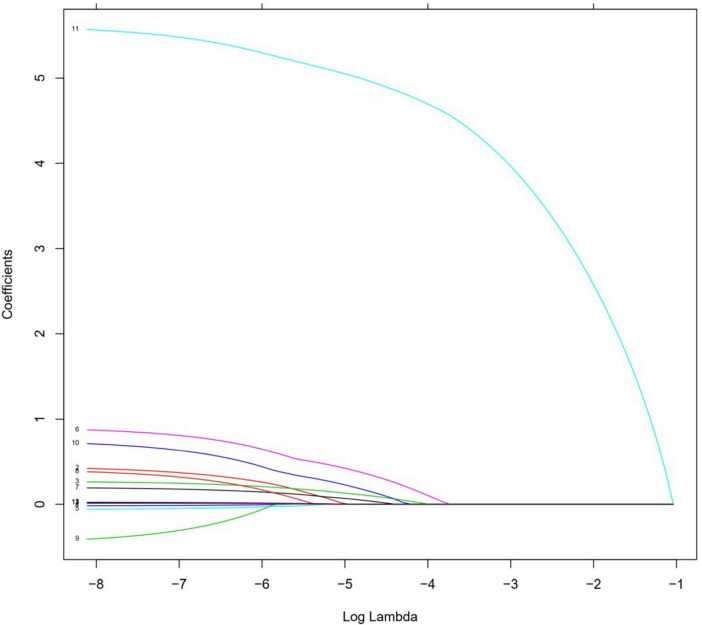
Lasso regression analysis of screening variables: screening retention variables when lambda is of optimal value.

Lasso regression controls the correlations between the screening variables by adjusting the lambda parameter, and the higher the value of lambda, the stronger the screening variables, and reducing the coefficients of non-characteristic variables to zero. The Lasso regression analysis showed that the optimal value of lambda was 0.008, which resulted in the best outcomes for the screened variables. The positive factors influencing patients’ post-discharge follow-up included occupation, interval.time, diagnosis, out.antipsychotics, and recommendations.followup, as illustrated in Figures 2, 3.

### 3.3. Construction of Nomogram prediction model

**FIGURE 4:**
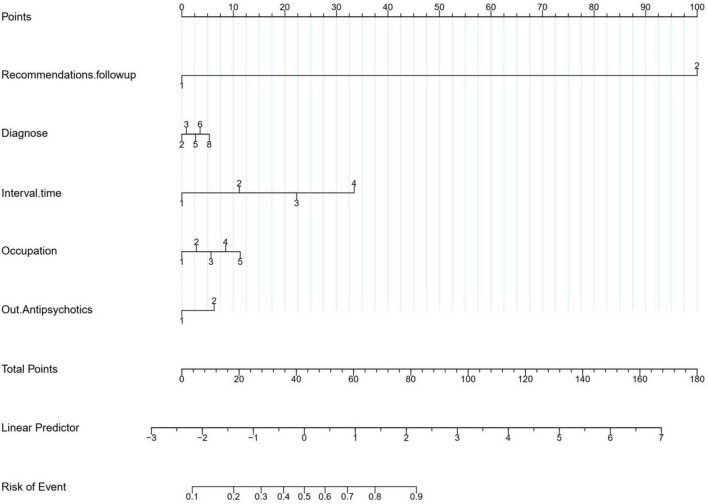
Nomogram prediction model diagram.

Lasso regression was applied to screen the variables for multi-factor logistic regression analysis, and the results showed that occupation, interval.time, diagnosis, out.antipsychotics, and recommendations.followup were independent positive factors influencing patients’ post-discharge follow-up, with no multicollinearity among the factors, as shown in Table 2. A Nomogram prediction model was constructed based on a weighted analysis of the regression coefficients of each independent positive factor. As shown in Figure 4, the predicted probability of the patients’ post-discharge psychosomatic follow-up was calculated by summing the scores of each independent positive factor in the Nomogram prediction model.

**TABLE 2:** Results of Logistics regression analysis.

Item	Estimate	Std.	Z value	*P*
Recommendations. followup	5.613	0.536	10.464	0
Diagnosis	0.05	0.148	0.336	0.737
Interval.time	0.626	0.341	1.837	0.066
Occupation	0.159	0.138	1.154	0.249
Out.antipsychotics	0.352	0.453	0.777	0.437

### 3.4. Evaluation of Nomogram prediction model

**FIGURE 5:**
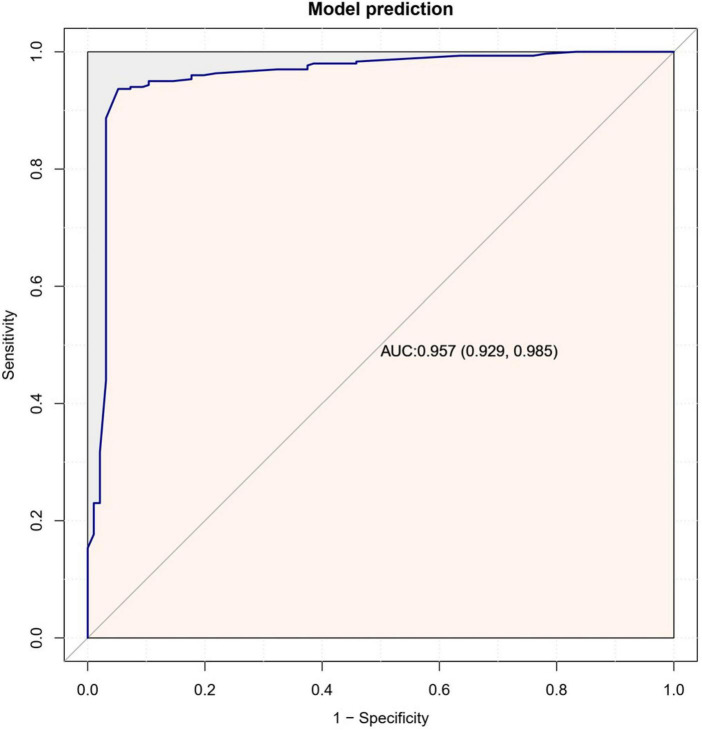
Receiver operating characteristic curve of patients who went for psychosomatic follow-up.

**FIGURE 6:**
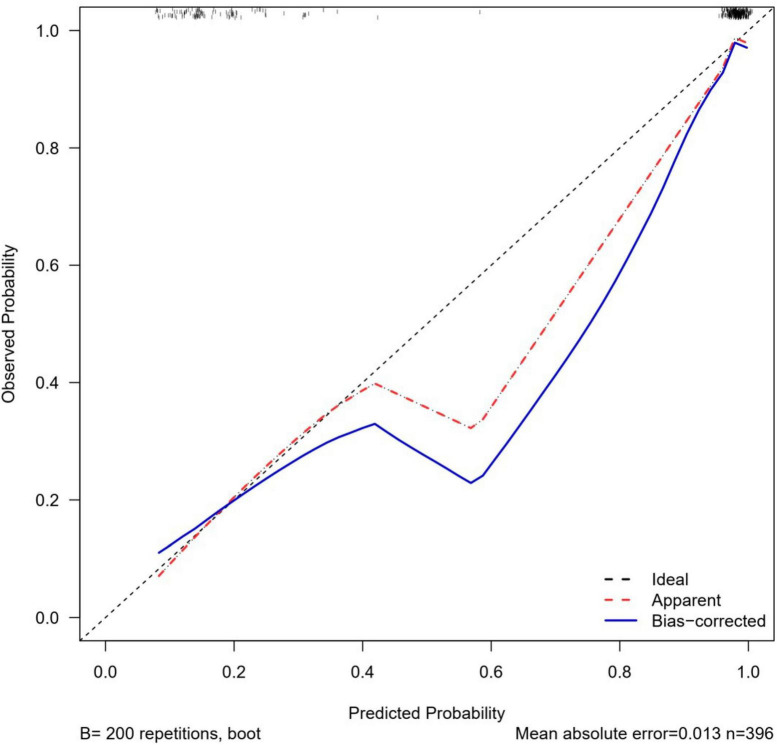
Nomogram prediction model calibration curve.

The Nomogram prediction model had a consistency index (C-index) of 0.914, and after 1,000 Bootstrap self-samplings, the C-index was 0.896. The area under the ROC curve was 0.957 (see Figure 5). As shown in Figure 6, the calibration curve (solid line in the figure) was positioned at the level of the diagonal dashed line with a slope equal to 1, close to the internal validation mark of the prediction model reached using Bootstrap; the C-index was 93.8 (87.5;100.0), and the bias-corrected curve was closer to the ideal reference line, indicating that the Nomogram prediction model had good calibration, discrimination, and risk prediction ability, and a comparable level of prediction.

### 3.5. Clinical utility of the Nomogram prediction model

**FIGURE 7:**
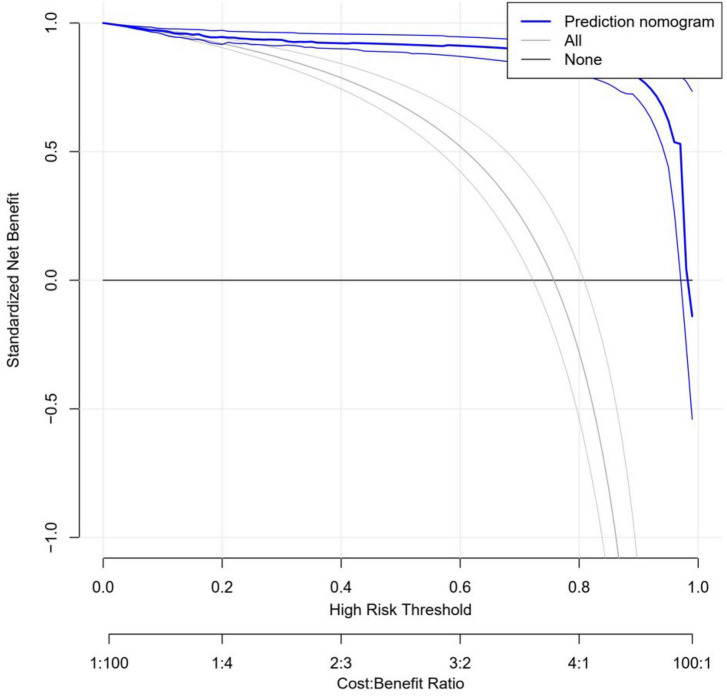
Nomogram prediction model clinical decision curve.

Figure 7 shows the results of the clinical decision curve analysis: the horizontal coordinate is the threshold probability and the vertical coordinate indicates the net benefit to the patient. The horizontal line indicates the positive factor effect of patients who did not obtain a follow-up visit when the relevant predictor and intervention were not given; the other curve is the positive effect of patients who underwent a follow-up visit after receiving the predictor-related factor intervention.; the blue curve shows the positive effect when the patient undergoes prediction-related factor intervention.

## 4. Discussion

In this study, the patients who received psychiatric liaison consultations had a mean age of 58.3 years, higher than that reported in a nationwide study (43.1–51.51 years) (5–8). As body systems decline to function with age, the elderly become susceptible to physical illnesses, including secondary or co-morbid psychiatric disorders. Furthermore, the present study showed that patients who went for follow-ups were significantly younger than patients who did not obtain a follow-up. This result may be related to patients’ awareness level of mental illness; younger people who may have received science education and are more aware of mental health take mental illness more seriously and accept it to a greater extent. The finding could also be attributed to patients’ occupations, which had a significant effect on whether patients went for follow-up visits (*p* < 0.05). Students had the highest percentage of follow-up visits. In a related study, the psychiatric follow-up rate in the adolescent inpatient group was 97.6% (9). Although the adolescent group accounted for a smaller number in the present study, the mean age was less and the follow-up rate was much higher than in other groups. There was no significant relationship between patients’ follow-up visits and whether they were from Xi’an or not, similar to the results of a related study (9), suggesting that the effect of the geographical location of the patient on the convenience of their access to care does not significantly affect their case access behavior.

There was also a significant difference in the proportion of patients opting for follow-up visits based on the interval of hospital stay: patients who went for follow-ups had significantly shorter intervals of hospital stay than those who did not come back for repeat visits (*p* < 0.05). Patients with longer hospitalization intervals were more depressed and anxious due to the increased treatment cost and the inability to return to everyday social life under economic and environmental pressure. A similar phenomenon was observed in patients hospitalized for prenatal pregnancy (10), pulmonary obstruction (11), and cardiovascular disease (12). A possible explanation is that after returning to their everyday living environment, depression and anxiety from long hospitalization stays dissipate, resulting in a lower follow-up rate. There was a significant difference (*p* < 0.05) in the percentage of follow-up visits depending on the interval between the time of admission and application for consultation. Patients who developed significant psychiatric symptoms during a short hospital stay often had psychosomatic problems before admission which were identified after admission to the hospital.

There was no significant difference in the overall patient follow-up rate regarding the reason for requesting a consultation (*p* > 0.05). In contrast, there was a significant difference in the follow-up rate of patients with different diagnoses (*p* < 0.05). Anxiety-depression disorders and schizophrenia/paranoid psychosis had the highest patient follow-up rate, suggesting that psychiatric liaison consultations in general hospitals are more oriented toward patients with non-organic psychiatric disorders.

There was no significant difference (*p* > 0.05) in the follow-up rate of patients who took medication or a combination of medication and psychological/physical treatment modalities. No significant difference was found in follow-up visits between patients considering whether or not they took medication during hospitalization (*p* > 0.05). However, there was a significant difference in follow-up visits of patients who were discharged with or without prescribed psychiatric medications (*p* < 0.05). The study found that patients’ attitude toward medication was a key factor influencing their follow-up rate, while patient compliance with treatment was negatively correlated with cognitive-psychological responses and positively correlated with patients’ trust in their psychiatrist (13). Some patients may prefer psychological counseling to medication (14). The more positive the attitude toward medicine, the higher the follow-up rate and the better the probability of using mood stabilizers during hospitalization (9).

There was also a significant difference (*p* < 0.001) in the follow-up of patients with and without a clear written medical order recommending a follow-up visit. Wang and Wang (15) suggest that annual household income per capita, duration of psychiatric examination, and written recommendations for discharge, including instructions on medication, influence the compliance of inpatients suffering from anxiety and depression in taking psychiatric medication in general hospitals.

Often, a semi-open interview is needed during the relatively short time between consultations to gather information about the impact of physical illness on the patient, the patient’s feelings about hospitalization, if the patient is confident in the treatment, and if there is any conflict between the patient and the supervising physician or healthcare provider. Many patients go through a difficult situation during the consultations and may show signs of regression and want to be understood (16).

Psychodynamic interpretation of observed patient cognitions and behaviors plays an important role in psychiatric liaison consultations. The psychiatric liaison consultant should observe patients’ attitudes and choose appropriate diagnostic language, paying attention to the cultural meanings of statements (17). Communication with patients of different ages should focus on using appropriate communication methods and approaches to avoid negative confrontation or denial of treatment by patients due to the diagnostic terminology. When patients fail to understand their experiences of loss, helplessness, hopelessness, and despair (18), they are more likely to accept the physicians’ treatment recommendations and opt for out-of-hospital psychiatric follow-up and intervention where they feel understood and supported (19). At the end of the consultation, a more precise medication regimen should be prescribed based on the evaluation of the patients’ feelings, which can promote medication adherence. Written communication for the discharge of patients should include detailed medication instructions and clear recommendations for the follow-up to accommodate patients in anxious and depressed states with weakened cognitive function. Taking time to build a trusting relationship during consultations provides patients with an atmosphere to safely and comfortably express their internal feelings and establish a good therapeutic alliance, thus improving patient compliance with follow-up visits (20).

## 5. Conclusion

In this study, patients who received a psychiatric liaison consultation with a ≤ 10-day interval between admission to the hospital and application for consultation, were discharged with prescribed medication, and had a clear written medical order for a follow-up consultation had an increased probability of psychosomatic follow-up after discharge. Following working methods in CLP can effectively improve participants’ compliance with follow-up recommendations. First, in the consultation process, different methods of explaining and communicating psychosomatic diseases should be adopted according to the individual conditions of the participants. Second, prescribe a more concise drug use plan before the end of the consultation. Third, written down advice when the consulted participant is discharged includes detailed medication guidance and clear recommendations for follow-up visits, and a take certain amount of time explaining the content and establishing a trusting relationship between doctors and participants.

## 6. Limitations

There are some limitations of this study. First, the study data were collected from only one hospital with a small sample size. Second, there were disaggregated data on patient use of physical therapy versus psychotherapy. Finally, patients were prescribed medication for 1 month at the time of discharge, which limited the follow-up duration to 1 month, and the study had to set a short follow-up observation period. However, patients might not have been able to visit the hospital for the follow-up on time due to various reasons, and the follow-up rate might have increased over an extended period, which was not taken into consideration in the design of this study.

## Data availability statement

The original contributions presented in this study are included in this article/supplementary material, further inquiries can be directed to the corresponding author.

## Author contributions

All authors contributed to the conceptualization of the study and approved the submitted version.
